# Adverse drug reactions, particularly liver disorders, drive interruptions in anti‐tuberculosis treatment: A retrospective cohort study

**DOI:** 10.1002/bcp.70197

**Published:** 2025-08-11

**Authors:** Eleanor G. Dixon, Evita Biraua, Edvards Brencsēns, Vitālijs Pašuks, Vija Riekstina, Anete Šperberga, Marisa D. Muckian, James W. Dear, Liga Kuksa, Derek J. Sloan, Helen R. Stagg

**Affiliations:** ^1^ Usher Institute University of Edinburgh Edinburgh UK; ^2^ NIHR RIGHT4: Preventing Deaths from Acute Poisoning in Low‐and Middle‐Income Countries, Centre for Cardiovascular Science University of Edinburgh Edinburgh UK; ^3^ Riga East University Hospital, Centre of Tuberculosis and Lung Diseases Riga Latvia; ^4^ Department of Infectious Disease Epidemiology London School of Hygiene & Tropical Medicine London UK; ^5^ School of Medicine University of St Andrews St Andrews UK

**Keywords:** drug‐related side effects and adverse reactions, retrospective cohort study, treatment adherence and compliance, tuberculosis

## Abstract

**Aims:**

Adverse drug reactions (ADRs) are a key driver of missed doses of anti‐tuberculosis (TB) therapy. We aimed to determine the relative burden of ADR‐driven missed doses, the missed dose patterns associated with ADRs, and the association between specific ADRs and missed doses.

**Methods:**

In this retrospective cohort study, adults (≥18 years) who began the standard 6‐month drug‐sensitive anti‐TB regimen in an outpatient facility in Riga, Latvia (May 2015–September 2022) and missed at least one dose of treatment were included. Data were collected from medical records and observed therapy records. Missed doses were subdivided into early discontinuation or sporadically missed. Descriptive analyses and lasagne plots were used.

**Results:**

Across 174 patients, 54 (31.0%, CI: 24.2–37.9%) missed doses due to ADRs. Of 31 320 doses, 4217 (13.5%, CI: 13.1–13.9%) were missed, 20.9% (880/4217, CI: 19.6–22.1%) were due to ADRs. Eighteen (10.3%) of the 174 patients discontinued treatment early, two of which (11.1%) were due to ADRs. Doses missed due to ADRs caused longer yet less frequent periods of sporadic missed doses: 56.4% (479/849) of sporadic missed doses were 1 day in length *vs*. only 9.1% (7/77) for ADR‐related ones. Hepatobiliary disorders were the leading ADR group causing missed doses. Hepatobiliary ADRs caused long median durations of missed doses (median 15.0, CI: 13.0–22.0).

**Conclusion:**

Our study underscores the importance of ADRs as a cause of missed doses of treatment, particularly hepatobiliary disorders. Regimens that are less prone to ADRs and strong healthcare system support structures for patients with ADRs are required to minimize missed doses, reducing unfavourable outcomes.

**What is already known about this subject**

- Adverse drug reactions (ADRs) are a known risk factor for missed doses of anti‐tuberculosis (TB) therapy.
- Relationships between different missed dose patterns and specific ADRs are not known.

**What this study adds**

- ADRs cause longer yet less frequent periods of missed doses of anti‐TB therapy.
- Hepatobiliary, gastrointestinal and skin disorders are the ADRs causing the most missed doses of anti‐TB therapy.

## 1 INTRODUCTION

Over the last decade, tuberculosis (TB) has killed more individuals globally than any other single infectious agent—an estimated 1.25 million people in 2023 alone.^1^, ^2^ Of the 7.1 million people known to start treatment for TB annually, 12% of drug‐sensitive patients have unfavourable treatment outcomes.^2^, ^3^

Missing doses of treatment is thought to be one key cause of poor treatment outcomes; anti‐TB regimens may be poorly ‘forgiving’ of even small numbers of doses of treatment being missed.^4^, ^5^ A wide array of factors that range from regimen to healthcare system‐related issues are well known to drive missed doses.^6^ These include adverse drug reactions (ADRs), which can result in both missed doses of treatment without the agreement of the healthcare provider (and thus be within the technical definition of non‐adherence^7^), and with their agreement.^8^ Anti‐TB drugs are known to be associated with a wide array of ADRs, including various forms of hepatotoxicity, including drug‐induced liver injury (DILI).^9^, ^10^, ^11^

In our recent scoping review, we identified that over half of the papers that explored the causes of missed doses identified ADRs as a key driver.^8^ Amongst these studies, only 38% listed the specific ADRs causing missed doses, and none described the patterns of missed doses due to ADRs. For example, do ADRs result in patients stopping their treatment early and not returning for treatment (early discontinuation)^12^ and, if so, how early? Do ADRs result in sporadic missed doses during the treatment course^12^ and, if so, how long are these treatment gaps and when do they occur? Knowing this is critical for patient care, since individuals who sporadically miss individual doses of anti‐TB therapy pose different healthcare system challenges to patients who discontinue therapy early.

Due to the results of our scoping review, in this study we sought to determine the importance of ADRs as a driver of missed doses of anti‐TB treatment and how ADR‐driven missed doses manifested during treatment. As such, the objectives were:

1. Amongst patients who missed doses of treatment, determine the percentage who missed doses due to ADRs.
2. Determine the percentage of doses missed due to ADRs, and the missed dose patterns associated with such missed doses.
3. Document the association between specific ADRs and missed doses.

## 2 METHODS

### 2.1 Study design and population

A retrospective cohort study was conducted at the Centre for Tuberculosis and Lung Diseases (an outpatient facility) in Riga, Latvia. Eligible patients were identified through the facility registry and directly/video observed therapy (DOT/VOT) records. These patients were adults (≥18 years) with TB at any disease site and who were recorded as having missed at least some of the drugs within one or more doses of therapy. Patients must have started treatment on the World Health Organization (WHO) recommended 4HRZE/2HR regimen for drug‐sensitive TB: a six‐month regimen composed of isoniazid (H, INH), rifampicin (R, RMP), pyrazinamide (Z, PZA) and ethambutol (E, EMB) for 2 months followed by 4 months of INH and RMP.^13^ Patients were prescribed and administered drugs individually (fixed‐dose combination [FDC] pills were not used) and therapy was prescribed daily. Changes to the regimen during treatment were allowed. Full details of the eligibility criteria are outlined in Appendix S1, and more information on the drug regimen is given in Appendix S2.

### 2.2 Data collection and cleaning

All data were recorded in paper records held at the hospital and, barring the missed dose data, were digitalized for this study into Research Electronic Data Capture (REDCap), which was hosted on a server at the University of Edinburgh.^14^, ^15^ REDCap is a secure, web‐based software platform designed to support data capture for research studies, providing (1) an intuitive interface for validated data capture; (2) audit trails for tracking data manipulation and export procedures; (3) automated export procedures for seamless data downloads to common statistical packages; and (4) procedures for data integration and interoperability with external sources. Unless otherwise stated, all data extraction was done by one author and checked by another author. Data were cleaned and analysed in RStudio (R‐4‐2.2), as outlined in Appendix S3.

### 2.3 Missed dose data

DOT/VOT records documenting missed doses and taken doses were digitalized into Microsoft Excel (Appendix S3). In an extension to standard definitions of non‐adherence (doses not taken as agreed between the patient and healthcare provider^7^), this study also included doses missed due to ADRs where the healthcare provider and patient agreed that a dose should be missed. We thus refer to ‘missed doses’, rather than ‘non‐adherence’, throughout.

Missed doses were classified as either sporadic or early discontinuation using a standardized taxonomy^12^: early discontinuation refers to cessation of treatment before its planned end date; all other missed doses were sporadic. Doses were further classed as fully missed (no drugs taken—could be either early discontinuation or sporadic missed doses) or partially missed (some drugs taken—sporadic missed doses only). In our descriptive analyses, we used two variables: one that charted fully missed doses (‘fully’) and another that charted both partially or fully missed doses (‘partially/fully’).

### 2.4 ADR data

Doses missed due to ADRs were identified through DOT/VOT records (Appendix S4). There were some missed dose periods that contained both doses missed due to ADRs and doses that were missed for other reasons. In some instances, doses missed due to ADRs directly preceded a period in the patient's DOT/VOT record for which there was no subsequent information, i.e., early discontinuation. In these instances, the doses missed due to ADRs were counted as part of the period of early discontinuation, but it was not assumed that the entire period of early discontinuation was due to ADRs.

The type of ADR associated with each missed dose period and the seriousness of the ADR were extracted from medical notes by one of three native Latvian‐speaking authors (clinicians); 10% of patient data was checked in duplicate (details in Appendix S4). The standardization of the ADR terminology (using MedDRA classifications), grouping of ADRs by class organ system (COS), and recording of their seriousness^16^ is documented in Appendix S4.

### 2.5 Demographic and clinical data

Additional demographic and clinical data were collected from the clinic's TB registry and the patient card. Detailed site of disease data were classed as pulmonary, extrapulmonary, or pulmonary and extrapulmonary.

### 2.6 Data analyses

Analyses were conducted in RStudio (R‐4‐2.2). Only the first 180 doses of therapy were analysed (Appendix S2). The analyses are described in turn below. For the confidence intervals (CIs) around medians, we did not calculate a result when there were five or fewer data points. Where there were 6–30 data points, we used a conservative (to ensure at least 95% coverage) exact binomial method based on order statistics, allowing for asymmetric CIs. When more than 30 data points existed, we used the centile command in Stata, which used the binomial method that makes no assumptions about the underlying distribution of the variable.

#### 2.6.1 Missed doses due to any cause

The percentage of doses partially/fully missed and fully missed were summarized, as well as missed sporadically and due to early discontinuation. The number of individuals who discontinued treatment early and when they discontinued was summarized. Missed dose data were summarized in lasagne plots.^17^ For partially/fully sporadically missed doses, the median number and median length of missed dose periods per patient were summarized. The percentage of such missed dose periods that were one dose in length was summarized across the entire study population.

#### 2.6.2 Patients who missed doses due to ADRs

Within the cohort of individuals who partially/fully missed at least one dose of treatment, we calculated the percentage of patients who had partially/fully missed at least one dose due to ADRs. Results were also presented per quartile of age across individuals included in the study.

#### 2.6.3 Doses missed due to ADRs

Analyses of ‘Missed doses due to any cause’ were repeated, only for doses specifically missed due to ADRs. Again, results were presented stratified by age.

#### 2.6.4 Doses missed due to specific ADRs

The ADRs named in the dataset as causing partially/fully missed doses were summarized, including their seriousness,^16^ the percentage of doses missed due to the ADR and the length of missed dose periods due to the ADR (Appendix S4 provides further details). The patterns of missed doses due to a given ADR were summarized in lasagne plots.

### 2.7 Ethics

Ethical approval was given by the Edinburgh Medical School Research Ethics Committee (EMREC), University of Edinburgh (23‐EMREC‐003) and the Medical and Biomedical Petitions Ethics Committee, Riga East Clinical University Hospital (18‐A/23).

## 3 RESULTS

A total of 174 patients were included in the cohort (Appendix S5); their demographic and clinical characteristics are presented in Table 1. A greater percentage of individuals with solely extrapulmonary disease missed doses due to ADRs (8/17, 47.1%) than people with other sites of disease. Patients 70–79‐year‐olds (6/11, 54.5%) and 50–59‐year‐olds (9/22, 40.9%) were the two age groups in which the greatest percentage of missed doses due to ADRs occurred.

**TABLE 1:** Demographic and clinical characteristics of patients.

	Patients		Patients with ≥1 partially/fully missed dose due to ADRs	
	Number	Column %	Number	Row %
**Sex**				
Male	116	66.7	31	26.7
Female	58	33.3	23	39.7
Unknown	0	0.0	0	0.0
**Age (years)**				
18–29	17	9.8	4	23.5
30–39	47	27.0	11	23.4
40–49	46	26.4	16	34.8
50–59	22	12.6	9	40.9
60–69	26	14.9	7	26.9
70–79	11	6.3	6	54.5
80+	5	2.9	1	20.0
Unknown	0	0.0	0	0.0
**Country of birth**				
Latvia	152	87.4	48	31.6
Other^a^	12	6.9	4	33.3
Unknown	10	5.7	2	20.0
**Site of disease**				
Pulmonary	134	77.0	40	29.9
Extrapulmonary^b^	17	9.8	8	47.1
Both	22	12.6	6	27.3
Unknown	1	0.6	0	0.0
**HIV status**				
Positive	32	18.4	11	34.4
Negative	125	71.8	41	32.8
Unknown	17	9.8	2	11.8

Abbreviations: ADR, adverse drug reaction; HIV, human immunodeficiency disease.

^a^ In addition to Latvia, six other countries of birth were identified, each with ≤5 patients.

^b^ Eleven different extrapulmonary disease sites were identified including TB of the bones and joints, genitourinary system and peripheral lymphadenopathy.

### 3.1 Missed doses due to any cause

Out of a total of 31 320 doses in the dataset, 4217 (13.5%, CI: 13.1–13.9%) were partially/fully missed, of which 3634 (86.2%) were fully missed. 80.6% (3399/4217) were partially/fully missed sporadically and 19.4% (818/4217) were fully missed due to early discontinuation. Eighteen patients discontinued their treatment early, with the median point of discontinuation being at dose 167, the earliest being dose 17 and the latest being dose 180.

**FIGURE 1:**
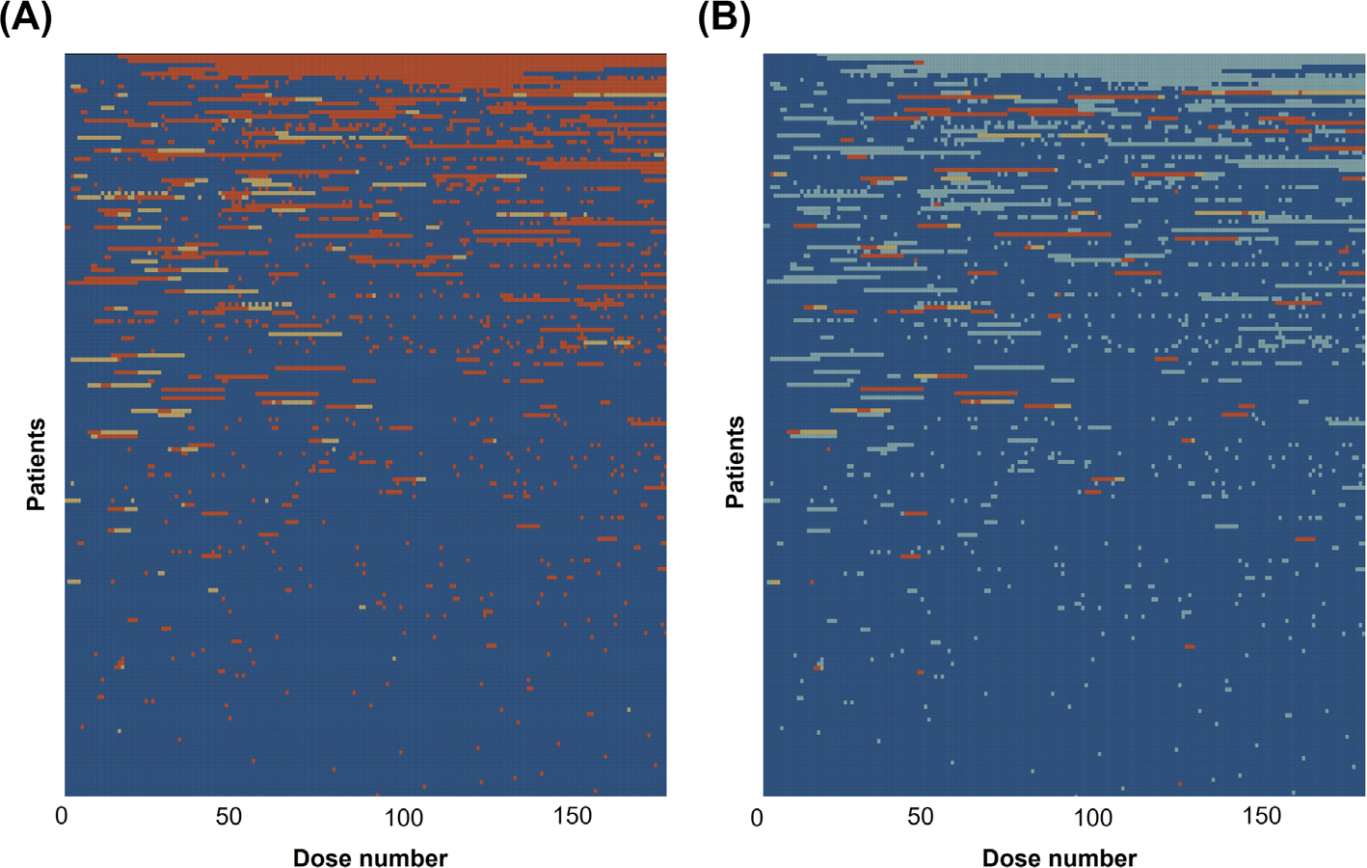
Lasagne plots of missed doses of treatment. Within these lasagne plots,^17^ patients are ordered top to bottom from least to most adherent (based on the percentage of partially/fully doses missed), with each patient represented as a row. On the x‐axis, we move through treatment from left (dose 1) to right (dose 180). The colours represent whether or how doses of treatment were missed. (A) overall picture of fully and partially missed doses. Dark blue, dose not missed. Orange, dose fully missed, i.e., all drugs. Yellow, dose partially missed (i.e., some but not all drugs). (B) missed doses due to ADRs. Dark blue, dose not missed. Light blue, doses missed not due to ADRs. Orange, dose fully missed due to ADRs. Yellow, dose partially missed due to ADRs. Note, more than 18 patients may appear from these plots to have discontinued early, as, for some individuals, treatment continued beyond 180 doses.

Lasagne plots of partially and fully missed doses indicated that doses were missed throughout treatment, with no clear pattern (Figure 1A).

**FIGURE 2:**
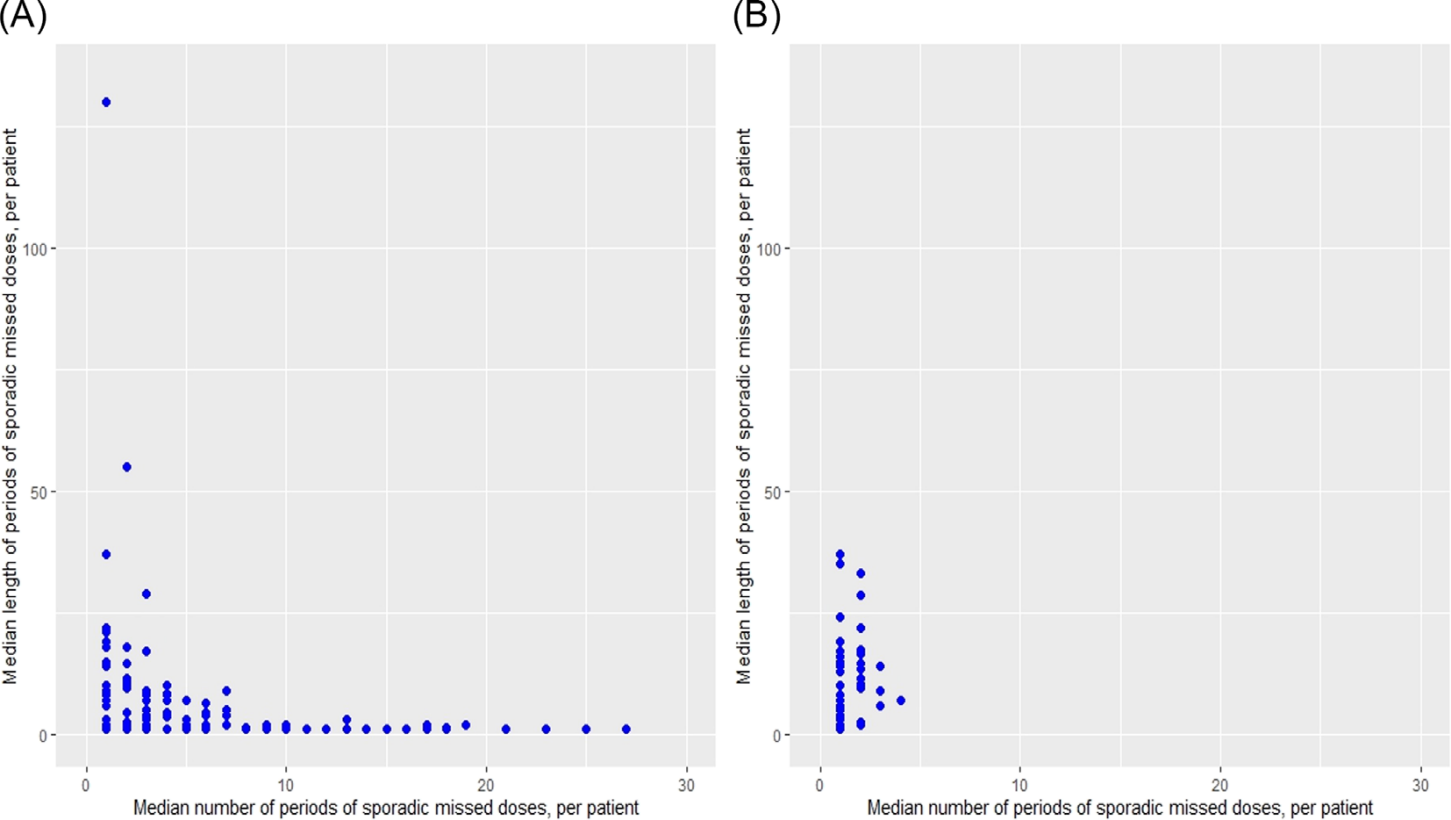
Periods of sporadic missed doses. The number of periods of sporadic missed doses per patient were plotted against the median length of missed dose periods for that patient. Both graphs have the same axis measurements to facilitate comparison between missed dose causes. (A) partially/fully missed doses due to any cause. (B) partially/fully missed dose due to ADRs.

Examining specifically sporadic missed doses (partially/fully missed), we detected a median of three (CI: 2–3) missed dose periods per patient (Figure 2A). The median length of the sporadic partially/fully missed dose periods per patient was 1.5 (CI: 1–2). In total, 56.4% (479/849) of sporadically partially/fully missed dose periods were 1 day in length.

### 3.2 Patients who missed doses due to ADRs

Fifty‐four patients (31.0%, CI: 24.2–37.9%) partially/fully missed at least one dose of treatment due to ADRs; of these, 53 patients (98.1%) fully missed at least one dose due to ADRs. By quartile of age, the percentage of people who partially/fully missed at least one dose due to ADRs for age group 18–34 years was 10/43 (23.3%, CI: 10.6–35.9%), 34–42 years was 11/44 (25.0%, CI: 12.2–37.8%), 43–58 years was 19/44 (43.2%, CI: 28.5–57.8%) and 58–91 years was 14/43 (32.6%, CI: 18.6–46.6%).

### 3.3 Doses missed due to ADRs

Of 4217 partially/fully missed doses, 880 (20.9%, CI: 19.6–22.1%) were missed due to ADRs. By quartile of age, the percentage of partially/fully missed doses due to ADRs for age group 18–34 years was 166/880 (18.9%, CI: 16.3–21.4%), 34–42 years was 202/880 (23.0%, CI: 20.2–25.7%), 43–58 years was 260/880 (29.5%, CI: 26.5–33.0%) and 58–91 years was 252/880 (28.6%, CI: 25.6–31.6%).

Of 3634 fully missed doses, 679 (18.7%) were missed due to ADRs. Of the 3399 partially/fully sporadically missed doses, 869 (25.6%) were due to ADRs and 11/818 (1.3%) fully missed doses due to early discontinuation were due to ADRs. Of the 18 individuals who discontinued their treatment early, 2 (11.1%) did so due to ADRs, at doses 45 (i.e., in the intensive phase) and 172 (i.e., nearly at the end of treatment) (median 109).

As per the data for doses missed due to any cause, doses missed due to ADRs were also spread throughout treatment (Figure 1B).

Sporadic missed dose periods (partially/fully missed) due to ADRs were less frequent but longer in duration than those due to any reason. We detected a median of one (CI: 1–1) sporadic missed dose period per patient (Figure 2B). The median length of a sporadic missed dose period due to ADRs per patient was 9 days (CI: 6–14). In total, 9.1% (7/77) of sporadically partially/fully missed dose periods were 1 day in length i.e., far fewer than when examining doses missed due to any reason.

### 3.4 Doses missed due to specific ADRs

Twenty‐three different ADRs causing partially/fully missed doses sporadically and at the start of early discontinuation were reported a total of 98 times. Of these, 29.6% (29/98) occurred concurrently. The most common concurrent ADRs were multiple hepatobiliary disorders (41.4%, 12/29), skin and subcutaneous disorders with immune system disorders (10.3%, 3/29) and multiple skin and subcutaneous disorders (10.3%, 3/29). There were a further 24 periods of missed doses due to ADRs where the ADR was unknown.

Out of all ADRs recorded, elevated liver enzyme levels, hepatotoxicity and hepatitis caused the most doses to be partially/fully missed, accounting for 57.3% (CI: 54.0–60.5%, 504/880), 25.8% (CI: 22.9–28.7%, 227/880) and 16.9% (CI: 14.5–19.4%, 149/880) of doses partially/fully missed due to ADRs, respectively; details of all ADRs recorded are presented in Table 2. This resulted in hepatobiliary disorders dominating the class organ system (COS) data, followed by gastrointestinal disorders and skin and subcutaneous tissue disorders. It is likely that concurrently observed ADRs were symptoms of the same underlying toxicity. For example, liver toxicity can cause symptoms across multiple COS.^18^, ^19^, ^20^

**TABLE 2:** Missed doses due to ADRs.

COS	ADR	Number of doses missed due to this ADR^a^	% of doses missed due to ADRs missed due to this ADR^b^ (CI)	Median length of missed dose period due to ADR^c^
Hepatobiliary disorders	Elevated liver enzyme levels	504	57.3 (54.0–60.5)	14.0
Hepatobiliary disorders	Hepatotoxicity	227	25.8 (22.9–28.7)	14.0
N/A	Unknown	191	21.7 (19.0–24.4)	6.5
Hepatobiliary disorders	Hepatitis	149	16.9 (14.5–19.4)	13.0
Hepatobiliary disorders	Cholestasis	85	9.7 (7.7–11.6)	13.0
Gastrointestinal disorders	Nausea	59	6.7 (5.1–8.4)	8.0
Skin and subcutaneous tissue disorders	Rash	54	6.1 (4.6–7.7)	5.0
Gastrointestinal disorders	Abdominal pain	53	6.0 (4.5–7.6)	53.0^d^
Skin and subcutaneous tissue disorders	Pruritus	48	5.5 (4.0–7.0)	7.0
Immune system disorders	Skin reactions	29	3.3 (2.1–4.5)	4.0
Gastrointestinal disorders	Vomiting	28	3.2 (2.0–4.3)	8.0
Blood and lymphatic system disorders	Thrombocytopenia	16	1.8 (0.9–2.7)	6.0
General disorders and administration site conditions	Pyrexia	16	1.8 (0.9–2.7)	8.0
Metabolism and nutrition disorders	Decreased appetite	15	1.7 (0.9–2.6)	15.0^d^
General disorders and administration site conditions	Asthenia	13	1.5 (0.7–2.3)	4.0
Gastrointestinal disorders	Dyspepsia	9	1.0 (0.4–1.7)	9.0^d^
Gastrointestinal disorders	Flatulence	9	1.0 (0.4–1.7)	9.0^d^
Blood and lymphatic system disorders	Lymphadenopathy	9	1.0 (0.4–1.7)	9.0^d^
General disorders and administration site conditions	Febrile disorders	8	0.9 (0.3–1.5)	8.0^d^
Gastrointestinal disorders	Acute pancreatitis	4	0.5 (0.0–0.9)	4.0^d^
Psychiatric disorders	Somnolence	4	0.5 (0.0–0.9)	4.0^d^
Immune system disorders	Urticaria	3	0.3 (0.0–0.7)	3.0^d^
Musculoskeletal and connective tissue disorders	Arthralgia	2	0.2 (0.0–0.5)	2.0^d^
Cardiac disorders	Paroxysmal tachycardia (supraventricular)	2	0.2 (0.0–0.5)	2.0^d^

*Note*: The number of doses missed due to individual ADRs and the median duration of the missed dose period due to each ADR. Confidence intervals (95%, CI) are given. Where this could not be calculated, NA is given. ADRs are ordered from the greatest contributors of missed doses to the least.

Abbreviations: ADR, adverse drug reaction; CI, 95% confidence interval; COS, class organ system; DOT, directly observed therapy; VOT, video observed therapy.

^a^ The number of doses missed due to ADRs within the table totals 1537 doses, which exceeds the total number of doses missed due to ADRs (880 doses); this is because some doses were missed due to multiple, simultaneous ADRs.

^b^ Denominator 880 doses.

^c^ For doses missed due to ADRs at the start of early discontinuation, the length of that missed dose period equals the number of doses recorded as missed due to ADRs on the patient's DOT/VOT records prior to there being no subsequent information.

^d^ Where there was only one missed dose period due to the ADR, the median represents the actual length of the missed dose period.

The two patients who discontinued treatment early due to ADRs did so due to cholestasis, elevated liver enzyme levels, hepatitis and hepatoxicity (patient one), and cholestasis and elevated liver enzyme levels (patient two).

Amongst COS where CIs were calculable (gastrointestinal disorders, hepatobiliary disorders, immune system disorders, skin and subcutaneous tissue disorders, unknown), hepatobiliary disorders had the largest median missed dose period, which was 15.0 missed doses (CI: 13.0–22.0). The next largest medians were for unknown COS (median 6.5 [CI: 3.0–11.0]) and gastrointestinal disorders (median 6.5 [CI: 1.0–21.0]).

Six patients experienced a total of seven (7.1%, 7/98) serious ADRs over six periods of missed doses; one patient had two concurrent serious hepatobiliary disorders causing missed doses. Of the serious ADRs which caused doses to be partially/fully missed, six (75.0%) were hepatobiliary disorders and one (12.5%) was blood and lymphatic disorders; all seven ADRs resulted in hospitalization. There was an additional serious ADR where the ADR was unknown, which also resulted in hospitalization.

**FIGURE 3:**
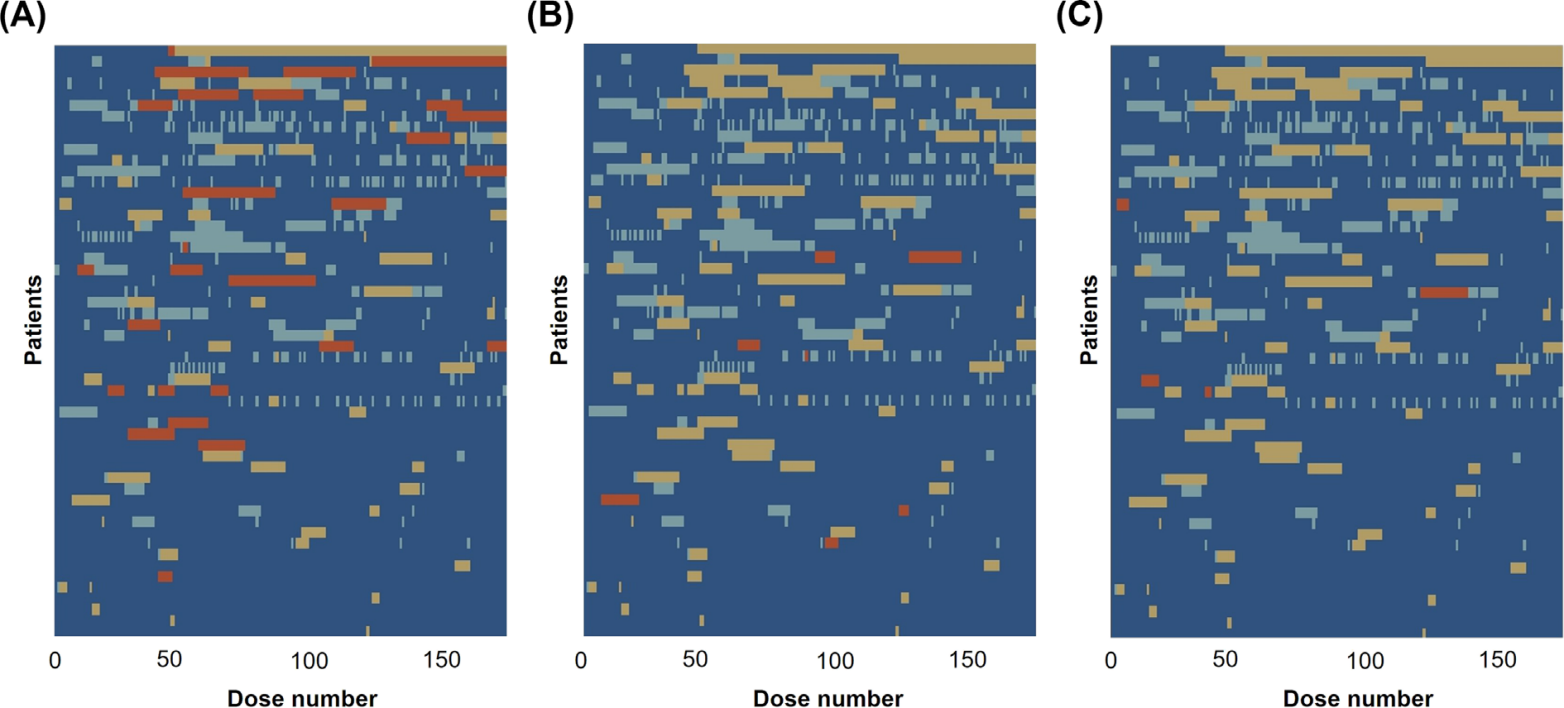
Missed doses due to ADRs. Lasagne plots include the 54 individuals who partially/fully missed doses due to ADRs. (A) doses missed due to hepatobiliary disorders. (B) doses missed due to gastrointestinal disorders. (C) doses missed due to skin and subcutaneous tissue disorders. Dark blue, dose not missed. Light blue, doses missed not due to ADRs. Orange, doses missed due to the plot‐specific ADR. Yellow, doses missed due to any other ADR. Patients are ordered on the plots based on the percentage of partially/fully doses missed (from greatest to least). Note, more than two patients may appear from these plots to have discontinued early due to ADRs, as, for some individuals, treatment continued beyond 180 doses.

Examining the lasagne plots of partially/fully missed doses by ADR (Figure 3), individuals missing doses due to skin and subcutaneous disorders (6/174 [3.4%] patients) did so primarily during the initiation phase of treatment. However, ADRs such as hepatobiliary disorders (24/174 [13.8%] patients) and gastrointestinal disorders (10/174 [5.7%] patients) occurred throughout therapy, with most occurring during the continuation period.

## 4 DISCUSSION

Building on the findings of our previous scoping review, this is the first study analysing patterns of missed doses of anti‐TB therapy due to ADRs.^8^ Approximately one‐third of patients who partially/fully missed doses of anti‐TB treatment in our cohort did so because of ADRs, which accounted for nearly a quarter of all sporadic partially/fully missed doses across the study cohort. Partial/full doses missed (sporadic and those at the start of early discontinuation) due to ADRs were spread throughout treatment. Patients who discontinued early tended not to sporadically miss large numbers of doses prior to discontinuing treatment. The median length of a partial/fully missed dose period (sporadic and start of early discontinuation) due to ADRs was greater than that of the partial/fully periods missed for any reason.

We report 23 types of ADRs causing missed doses spread across an array of COS, with hepatobiliary disorders both causing the greatest percentage of partially/fully missed doses and highest frequency of partial/fully missed dose periods, as well as long partial/fully missed dose periods.

We identified ADRs as an important driver of missed doses, consistent with our previous findings, potentially more so in older age groups.^8^ We found that hepatobiliary disorders, specifically elevated liver enzyme levels, hepatotoxicity and hepatitis, caused the most doses to be missed. This fits with three of the four drugs in the HRZE regimen being known hepatotoxic agents^21^, ^22^; however, the frequency of such ADRs is described as ‘unknown’, ‘uncommon’ or is not specified in these drugs' regulatory documentation.^23^, ^24^, ^25^, ^26^

Latvia is tackling a high prevalence of hepatitis C virus (HCV) infection (2.4%) and high rates of alcohol‐attributed morbidity and mortality.^27^ Some patients in the cohort are thus likely to have had compromised hepatobiliary systems increasing their vulnerability to hepatobiliary disorders during anti‐TB therapy.^28^, ^29^, ^30^ Indeed, a recent meta‐analysis found cause for recommending routine testing of HCV amongst all patients with TB due to the global seroprevalence of HCV and association with TB.^31^

The seriousness of hepatobiliary ADRs cannot be underestimated; one study identified that 23% of individuals (most of whom were HIV negative) with DILI from the HRZE regimen die.^22^, ^32^ With reports of DILI increasing each year,^33^ it is essential to provide ongoing support to patients experiencing ADRs—both at the initial presentation and throughout the following months of treatment—to ensure early detection and proactive management. For such ADRs, regular monitoring of liver enzymes has been suggested such that proactive changes to drug regimens can occur before ADR escalation leading to missed doses. However, there are doubts about the benefits of this for patients, given the need for regular invasive tests, and concerns about the implications for healthcare system costs, including staff resources.^34^ Clinicians could also engage in exploring protective drugs with patients upon or before ADR presentation (e.g. antiemetics).^35^ Still, some clinicians may be wary of introducing additional drugs into an already polypharmacy regimen, especially when there is doubt over the effectiveness of protective agents in often multimorbid patients.^33^

We present the first analysis of missed dose patterns associated with ADRs using granular dose taking data, including the ability to examine both partially/fully and fully missed doses of treatment. Although the granularity of the data did facilitate this analysis, most patients globally administer HRZE anti‐TB therapy as FDC pills, rather than individual drug tablets.^36^ This may limit the generalizability of the results, as does the background high prevalence of HCV, elevated alcohol consumption, but low HIV prevalence in this cohort. In a Cochrane review comparing the efficacy, safety and acceptability of FDCs compared to single‐drug formulations, outcomes (including relating to treatment dose taking and adverse events) between the two formulations were comparable.^36^ Moreover, most doses were fully missed (86.2% of all doses and 80.1% of doses missed due to ADRs), suggesting that patterns of missed doses due to ADRs would not differ substantially if FDC pills are used. Saying this, single‐drug pills open up the option for patients to not take specific drugs if they attribute side effect symptoms to that drug.

Due to the scope of this study, we only captured ADRs which caused doses to be missed. We were not able to capture the dates that ADRs started/finished from medical records, and thus our dataset was driven by when these ADRs became problematic enough to cause missed doses. ADRs that caused doses to be missed but which were not recorded in DOT/VOT records were not considered within our study, likely leading us to underestimate the proportion of doses that were missed due to ADRs. It is also likely that the number of doses missed due to ADRs during early discontinuation is higher than those presented, as we only attributed the first few missed doses where specific documentation was in place. The findings of our study can guide stakeholders who are intervening to minimize missing doses of therapy. Where strong support systems exist for early detection of ADRs and rapid action to help patients with them, the increased interaction between the patient and their healthcare provider has been shown to decrease missed doses.^37^, ^38^ Stakeholders must be aware that ADRs not viewed as clinically sufficient to pause treatment may still cause patients to struggle with dose‐taking, leading to an increase in sporadic missed doses for some or all drugs.^8^ Healthcare staff must be fully trained to proactively recognize possible ADRs and to act by balancing drug effectiveness with patient safety.^39^ Further understanding of what and how ADRs cause an increase or decrease in missed doses is essential in building effective, pragmatic strategies to minimize missed doses, maintain drug efficacy and ultimately reduce unfavourable outcomes from anti‐TB treatment. It will be essential to seek patient perspectives and assess local resources when implementing such strategies.^40^

In our study of a cohort of individuals who partially/fully missed at least one dose of drug‐sensitive TB treatment in Latvia, nearly a third missed doses due to ADRs, and ADRs caused nearly a quarter of all sporadic missed doses. ADRs caused doses to be sporadically missed for longer periods than doses missed for other reasons, with hepatobiliary disorders being a major cause of sporadically missed doses. Regimens that are less prone to ADRs and strong healthcare system support structures for patients with ADRs are required to minimize missed doses, reducing unfavourable outcomes and mortality.

## AUTHOR CONTRIBUTIONS

E.G.D., J.W.D., L.K., D.J.S. and H.R.S. designed the study. E.G.D., Ev.B., Ed.B., V.P., V.R. and A.S. acquired data for the study. E.G.D., M.D.M. and H.R.S. analysed the data for the study. All authors were involved in the writing or reviewing of the manuscript. All authors approved the final manuscript.

## CONFLICT OF INTEREST STATEMENT

The authors do not have any conflicts of interest to declare.

## Supporting information


**Table S1:** Study eligibility criteria.
**Table S2:** Coding of the missed dose data.
**Table S3:** Standardization of ADR language.
**Figure S1:** Study participants' selection.

## Data Availability

A copy of the cleaned dataset can be obtained upon request, and subject to approval, by contacting Helen Stagg (author) or Liga Kuksa (author).
