# Supplementary material for: Adverse drug reactions, particularly liver disorders, drive interruptions in anti‐tuberculosis treatment: A retrospective cohort study
Source: Br J Clin Pharmacol. 2025 Aug 11;91(12):3461–70. doi: 10.1002/bcp.70197 (PMC12648357; doi:10.1002/bcp.70197)
Supplement: Supplementary file 1 — Table S1: Study eligibility criteria. Table S2: Coding of the missed dose data. Table S3: Standardization of ADR language. Figure S1: Study participants' selection. [file BCP-91-3461-s001.docx]

# Appendix 1- Inclusion criteria

Only patients aged ≥18 years were included in the study as children do not autonomously take their medication. Drug susceptibility was assessed using Xpert MTB/ RIF ULTRA and/or culture methods and was measured once at the start of treatment. Daily adherence data (in the form of DOT/VOT) were required to identify patients who missed ≥1 dose of anti-TB therapy (see Missed dose data section of main manuscript for further clarification).

Patients were screened for eligibility from the hospital’s paper records. This was conducted in reverse chronology, starting with patients who initiated treatment in January 2022 (Appendix 5).

**Table S1: Study eligibility criteria |** TB (tuberculosis); 4HRZE/2HR (a six-month regimen composed of isoniazid (H, INH), rifampicin (R, RMP), pyrazinamide (Z, PZA) and ethambutol (E, EMB) for two months followed by four months of INH and RMP).

| **Inclusion** | **Exclusion** |
| --- | --- |
| 1. Active TB (bacteriologically confirmed by culture or GeneXpert). 2. Any site of disease. 3. Age ≥18 years. 4. Drug-sensitive TB (TB bacteria not resistant to RIF). 5. Started treatment on 4HRZE/2HR regimen, dosed daily (therapy could be changed during treatment). 6. Had daily adherence data. 7. Missed at least some of the drugs within ≥1 dose of anti-TB medication. | 1. No microbiological confirmation of active TB. 2. Age <18 years. 3. Not drug-sensitive TB (drug-resistant to RIF). 4. Did not start treatment on the 4HRZE/2HR regimen. 5. Did not have daily adherence data. 6. Did not at least miss some of the drugs within ≥1 dose of anti-TB medication. |

# Appendix 2- Drug regimen

Patients were prescribed and administered drugs individually- FDC pills were not used. Most patients started on a total of ten tablets per dose:

- Isoniazid: 300mg – one tablet
- Rifampicin: 600mg – two tablets
- Ethambutol: 1200mg – three tablets
- Pyrazinamide: 2000mg – four tablets

Patient dose was adjusted according to weight. If the patient’s weight was ≥50kg, the standard dose was given (as above). If the patient weighed <50kg, the drug dosage was adjusted accordingly.

At the facility, ‘six months’ of therapy varied between six calendar months or 180 days. In our analysis, the first 180 days of treatment were analysed for all patients. Of the 174 patients, 158 (90.8%) had more than 180 days of treatment.

Therapy was prescribed daily, but at the study site DOT/VOT was not necessarily administered every day. Where a patient did not attend the study site, the reason for the missed dose was investigated. Details on data handling are present in Appendix 3.

# Appendix 3- Cleaning the missed dose data

DOT/VOT record data were coded as per Table S2. These codes were taken from those written in the patients’ DOT/VOT records, i.e., each scenario was recorded differently in the DOT/VOT records. Doses were considered taken if a member of staff witnessed the drug(s) being taken (DOT), the drug(s) were confirmed to have been taken at another healthcare facility, or the drug had been observed by a member of staff virtually (live video call i.e., VOT). Further, drugs could have been taken without observation, but with staff permission.

On some occasions- e.g., when the DOT/VOT clinic was closed or patients were away- patients were not prescribed a dose of treatment. As this was a clinical decision, such doses were not counted as missed within the dataset. The longest period of non-prescribed doses was five doses and the shortest was one dose.

Table S2: Coding of the missed dose data

Coding of the missed dose data directly taken from the DOT/VOT records. Two variables were generated for the analyses- fully missed doses (i.e., all drugs for a given dose were missed) and partially/fully missed doses (some or all drugs for a given dose were missed). Blue highlights the difference between the two variables. *The absence of data indicated that the patient’s record was not accessed on that day i.e., the patient did not turn up to clinic. DOT (directly observed therapy); VOT (video observed therapy). ^This is early discontinuation.

| **DOT/VOT data for the dose** | **Fully missed dose variable** | **Partially/fully missed dose variable** |
| --- | --- | --- |
| All drugs in the dose missed, for any reason | Dose missed | Dose missed |
| Some, but not all, drugs in the dose taken on-site | Dose not missed | Dose missed |
| Some, but not all, drugs in the dose taken off-site/unobserved | Dose not missed | Dose missed |
| All the drugs in the dose taken on-site | Dose not missed | Dose not missed |
| All the drugs in the dose taken off-site/unobserved | Dose not missed | Dose not missed |
| Dose not prescribed on that day | Dose not missed | Dose not missed |
| No information for any drugs, but information for doses later in treatment* | Dose missed | Dose missed |
| No information for any drugs and no information later in treatment*^ | Dose missed | Dose missed |

# Appendix 4- ADR data entry and cleaning

As part of routine clinical practice, doses missed due to ADRs are specifically labelled in patients’ DOT/VOT records, identifying them from doses missed for other reasons. This process uses medical histories and biochemical tests (e.g., blood tests) to ascertain causality (clinical judgement).

ADRs were extracted from patients’ medical notes by three clinicians. Ten percent of the patients’ data was checked in duplicate for transcription errors. No discrepancies across the collected data were found thus no further checking was required.

Consistent with other publications (1), ADR terminology was standardised using Medical Dictionary for Regulatory Activities (MedDRA) terminology (2). Common ADRs were given as controlled options within REDCap, with free text options available (3-6). MedDRA terminology (2) was assigned to free-text ADR terms to ensure a universal understanding of ADR terms.

ADRs identified as causing missed doses were subsequently categorised by class organ system (COS) (1); groupings are presented in Table S3. ADRs categorised under multiple MedDRA COS were assigned to a single COS based upon the most typically prominent symptoms of the ADR and the number of doses missed due to the ADR; this process was conducted in duplicate by two authors (EGD and JWD).

ADRs were considered serious if they met one of the following criteria: results in death, is life-threatening, requires hospitalisation or prolongation of existing hospitalisation, results in persistent or significant disability or incapacity, or is a birth defect (7).

Table S3: Standardisation of ADR language

Each ADR term was standardised using MedDRA preferred labels and categorised into COS. The ADRs are ordered alphabetically by COS. ADR- adverse drug reaction; COS- class organ system. MedDRA: medical dictionary for regulatory activities.

| **Source data** | **MedDRA preferred label** | **COS** |
| --- | --- | --- |
| Thrombocytopenia | Thrombocytopenia | Blood and lymphatic system disorders |
| Lymphadenopathy | Lymphadenopathy | Blood and lymphatic system disorders |
| Supraventricular paroxysmal tachycardia | Paroxysmal tachycardia (supraventricular) | Cardiac disorders |
| Acute pancreatitis | Pancreatitis acute | Gastrointestinal disorders |
| Abdominal pain | Abdominal pain | Gastrointestinal disorders |
| Nausea | Nausea | Gastrointestinal disorders |
| Vomiting | Vomiting | Gastrointestinal disorders |
| Dyspepsia | Dyspepsia | Gastrointestinal disorders |
| Flatulence | Flatulence | Gastrointestinal disorders |
| Asthenia | Asthenia | General disorders and administration site conditions |
| Febrility | Febrile disorders | General disorders and administration site conditions |
| Fever | Pyrexia | General disorders and administration site conditions |
| Cholestatic reactions | Cholestasis | Hepatobiliary disorders |
| Elevated liver function test | Elevated liver enzyme levels | Hepatobiliary disorders |
| Hepatitis | Hepatitis | Hepatobiliary disorders |
| Hepatotoxicity | Hepatotoxicity | Hepatobiliary disorders |
| Skin reactions | Skin reactions | Immune system disorders |
| Urticaria | Urticaria | Immune system disorders |
| Anorexia | Decreased appetite | Metabolism and nutrition disorders |
| Arthralgia | Arthralgia | Musculoskeletal and connective tissue disorders |
| Somnolence | Somnolence | Psychiatric disorders |
| Pruritus | Pruritus | Skin and subcutaneous tissue disorders |
| Rash | Rash | Skin and subcutaneous tissue disorders |
| Unknown | Unknown | Unknown |

# Appendix 5- Participant flow diagram

Eligible patients were identified from the Centre for Tuberculosis and Lung Diseases’ registry and DOT/VOT records. Screening of patient records occurred in reverse chronological order, with patients registered at the facility most recently screened first; patient screening only occurred on patients who had started treatment during or before January 2022 to ensure full treatment records were present. The flow diagram of participants is presented in Figure S1.

**Figure S1: Study participants’ selection**

*A diagram illustrating the flow and identification of eligible patients.*


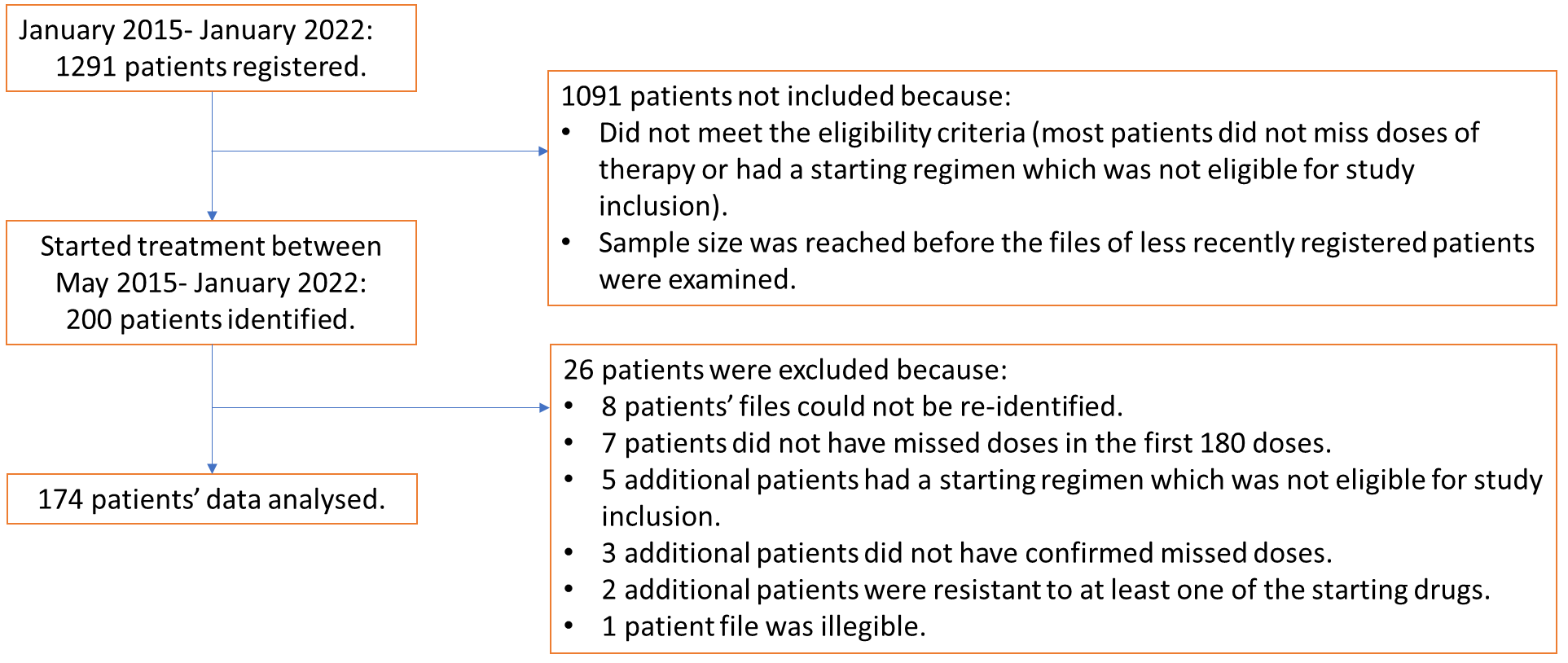


# References

1. Loke YK, Price D, Herxheimer A. Systematic reviews of adverse effects: framework for a structured approach. BMC Medical Research Methodology. 2007;7(1):32.

2. Medical Directory for Regulatory Activities. Medical Dictionary for Regulatory Activities Terminology (MedDRA) 2020 [updated 24/09/2020. Available from: <http://bioportal.bioontology.org/ontologies/MEDDRA>.

3. National Institute for Health and Care Excellence. Rifampicin <https://bnf.nice.org.uk/drugs/rifampicin/#side-effects2024> [Available from: <https://bnf.nice.org.uk/drugs/rifampicin/#side-effects>.

4. National Institute for Health and Care Excellence. Isoniazid <https://bnf.nice.org.uk/drugs/isoniazid/#side-effects2024> [Available from: <https://bnf.nice.org.uk/drugs/isoniazid/#side-effects>.

5. National Institute for Health and Care Excellence. Ethambutol 2024 [Available from: <https://bnf.nice.org.uk/drugs/ethambutol-hydrochloride/#side-effects>.

6. National Institute for Health and Care Excellence. Pyrazinamide 2024 [Available from: <https://bnf.nice.org.uk/drugs/pyrazinamide/#side-effects>.

7. European Medicines Agency. Serious adverse reaction 2024 [Available from: <https://www.ema.europa.eu/en/glossary-terms/serious-adverse-reaction#:~:text=An%20adverse%20reaction%20that%20results,or%20is%20a%20birth%20defect>.
